# Highly efficient *Agrobacterium rhizogenes*-mediated hairy root transformation for gene editing analysis in cotton

**DOI:** 10.3389/fpls.2022.1059404

**Published:** 2022-12-23

**Authors:** Lili Zhou, Yali Wang, Peilin Wang, Chunling Wang, Jiamin Wang, Xingfen Wang, Hongmei Cheng

**Affiliations:** ^1^ Biotechnology Research Institute, Chinese Academy of Agricultural Sciences, Beijing, China; ^2^ State Key Laboratory of North China Crop Improvement and Regulation, Hebei Agricultural University, Baoding, China; ^3^ National Nanfan Research Institute (Sanya), Chinese Academy of Agricultural Sciences, Sanya, China

**Keywords:** cotton, agrobacterium rhizogenes, hairy root, GhPDS, CRISPR/Cas9

## Abstract

CRIPSR/Cas9 gene editing system is an effective tool for genome modification in plants. Multiple target sites are usually designed and the effective target sites are selected for editing. Upland cotton (*Gossypium hirsutum* L., hereafter cotton) is allotetraploid and is commonly considered as difficult and inefficient to transform, it is important to select the effective target sites that could result in the ideal transgenic plants with the CRISPR-induced mutations. In this study, *Agrobacterium rhizogenes*-mediated hairy root method was optimized to detect the feasibility of the target sites designed in cotton phytoene desaturase (*GhPDS*) gene. *A. rhizogenes* showed the highest hairy root induction (30%) when the bacteria were cultured until OD_600_ reached to 0.8. This procedure was successfully applied to induce hairy roots in the other three cultivars (TM–1, Lumian–21, Zhongmian–49) and the mutations were detected in *GhPDS* induced by CRISPR/Cas9 system. Different degrees of base deletions at two sgRNAs (sgRNA5 and sgRNA10) designed in *GhPDS* were detected in R15 hairy roots. Furthermore, we obtained an albino transgenic cotton seeding containing CRISPR/Cas9-induced gene editing mutations in sgRNA10. The hairy root transformation system established in this study is sufficient for selecting sgRNAs in cotton, providing a technical basis for functional genomics research of cotton.

## 1 Introduction

Cotton is an important economic crop and natural fiber source (Huang and Zhu, 2021). In 2007, the Cotton Genome Consortium set a strategic plan to sequence cotton genomes (Chen et al., 2007). *Gossypium raimondii* was the first cotton species to be performed whole genome sequencing (WGS) (Paterson et al., 2012; Wang et al., 2012). The draft genome of *Gossypium arboreum* was released in 2014 (Li et al., 2014). In 2015, the draft genome of *Gossypium hirsutum* cultivar TM–1 was released (Li et al., 2015; Zhang et al., 2015). Over the next three years, new sequencing platforms and assembly strategies were used to further refine the upland cotton genome. In the same year, *Gossypium barbadense* genome was also sequenced (Liu et al., 2015; Yuan et al., 2015). In the next few years, a large amount of genomic information has been obtained by sequencing and resequencing, which provides a good foundation for functional genomics research.

The identification of gene function is usually achieved by overexpression and knockout. CRISPR/Cas9 has been developed into a recognized gene editing tool because of its wide applicability and diverse editing types. Compared with other genome-editing systems, CRISPR/Cas9 system has obvious advantages such as simple construction, low cost and easy implementation in the laboratory, so it has been widely used in genome editing of many plants (Jiang et al., 2013; Brooks et al., 2014; Raffan et al., 2021) and has shown great application prospects in cotton functional genomic research (Wang et al., 2017; Janga et al., 2019; Gao et al., 2020). Obtaining transgenic plants is particularly important for gene function research. For cotton transformation, it is particularly difficult to obtain transgenic plants from regeneration cultures using traditional plant transformation methods (Trolinder and Goodin, 1987; Vijaya et al., 2015). It typically takes about one year to obtain a transgenic cotton plant. Therefore, the reliability of the vector used for the transformation is particularly important.

sgRNA is one of the important elements of CRISPR/Cas9 system. Previous studies have shown that many sgRNAs do not work effectively (Farboud and Meyer, 2015). Therefore, the selection and editing efficiency of target sites are important issues to be considered before stable transformation. Transient transformation of protoplast and cotyledon with complete editing vectors are the two reported methods for selecting sgRNAs (Chen et al., 2017; Gao et al., 2017). However, due to the low transient transformation efficiency and the large size of the *Cas9* gene (more than 4000 bp), the gene editing event is not easy to be detected.

*Agrobacterium rhizogenes* (*Rhizobiaceae*), a gram-negative soil bacterium, contains the Ri plasmid and could induce hairy roots production from plants (Smith and Townsend, 1907). Since hairy roots have the advantages of rapid growth and multiple branches, it is easier to perform the induction of hairy roots using *A. rhizogenes* than to generate transgenic plants using *Agrobacterium tumefaciens*. *A. rhizogenes*-mediated transformation system has been established to test the efficiency of zinc-finger nucleases (ZFNs) and CRISPR/Cas9 construct in soybean (Curtin et al., 2011; Cheng et al., 2019) and other plants (Gai et al., 2015; Zhang et al., 2020). However, the validation of sgRNAs for CRISPR/Cas9 in cotton by *A. rhizogenes*-mediated hairy root system has not been reported yet. In this study, we developed a cotton hairy root system to validate sgRNAs for CRISPR/Cas9 gene editing in cotton within one month, which provides a reliable reference for stable transformation. This method was successfully used for the validation of sgRNAs targeting *GhPDS* gene. Moreover, CRISPR/Cas9-induced mutations were detected at GhPDS-sgRNA10 target site in a transgenic albino cotton seeding.

## 2 Results

### 2.1 Hairy root induction in cotton using *A. rhizogenes*

**Figure 1:**
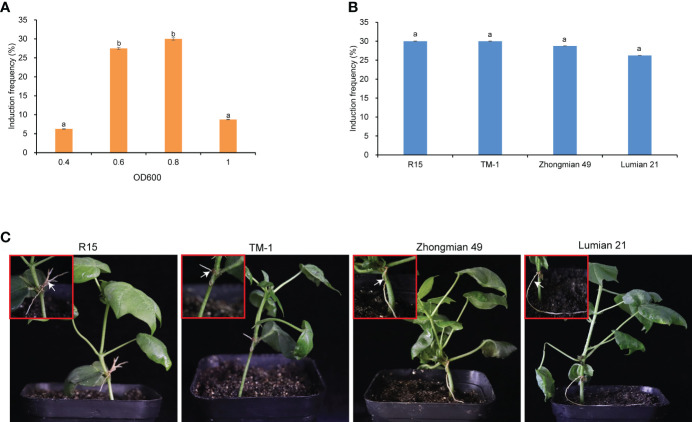
Induction of cotton hairy roots. **(A)** Comparison of hairy root induction frequency of *A rhizogenes* with different OD_600_ (0.4, 0.6, 0.8 and 1.0) in R15. **(B)** Hairy root induction frequency of *A rhi-zogenes* (OD_600_ 0.8) in four cotton cultivars (R15, TM-1, Zhongmian-49 and Lumian-21). Values are mean ± SD of four biological replicates, means with different letters denote a significance dif-ference while similar letters denote no significance (Tukey’s HSD test, p < 0.05). **(C)** Hairy root growth status in four cotton cultivars. Scale bars in c, 1.0cm.

To determine the optimal *A. rhizogenes* concentration for inducing hairy roots, a gradient optical density (OD_600_) experiment was conducted. R15 cotton seedings with the fully unfolded first true leaf were injected at the apical bud region with four OD_600_ (0.4, 0.6, 0.8, 1.0) suspensions of *A. rhizogenes*. Hairy roots emerged within one month, the ratio of R15 that produced hairy roots was calculated. The suspension of *A. rhizogenes* at OD_600_ 0.8 showed the highest induction frequency of 30%, followed by that at OD_600_ 0.6 (27.5%) (**Figure 1A**). This result indicated that OD_600_ 0.6–0.8 is the most suitable concentration range for inducing hairy roots. Later, we evaluated the procedure in the other three cultivars (Zhongmian–49, Lumian–21, TM–1). *A. rhizogenes* could induce hairy roots from these three cultivars and no significant difference in induction frequency of Zhongmian–49, Lumian–21, TM–1 and R15 when the bacteria were cultured to OD_600_ 0.8 (**Figure 1B, C**). These results indicated that this method is suitable for cotton materials with different genetic backgrounds.

### 2.2 CRISPR/Cas9 vector construction and target selection

**Figure 2:**
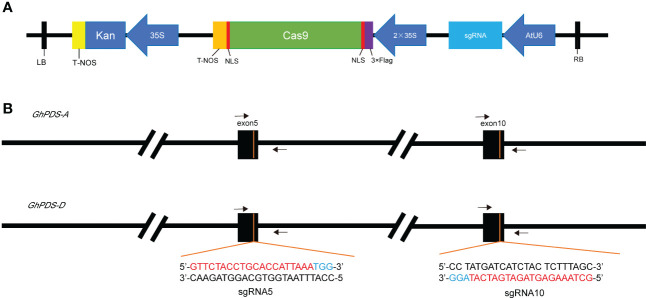
Diagram of single gRNA CRISPR/Cas9 vector, target sequence and target location. **(A)** CRISPR/Cas9 vector for cotton transformation with pCAMBIA2301 backbone, *kan* gene as selection marker for stable transformation, *Cas9* and sgRNA were driven by *CaMV*35S promoter and Arabidopsis U6 promoter respectively. NOS, Nopaline Synthase terminator. **(B)** Location of target sites in *GhPDS* in A genome and D genome and gene-specific primers used for sequencing were indicated by arrows.

The CRISPR vector used here encodes *Cas9* driven by the *CaMV*35S promoter and the sgRNA driven by the Arabidopsis U6 promoter (**Figure 2A**). Most genes in cotton are duplicated, so we designed the target sites that could target *GhPDS* in both A and D genomes simultaneously. sgRNA5 and sgRNA10 were designed to guide Cas9 to cleave the target site within the fifth and tenth exons of *GhPDS*, respectively, and the primer sites for amplification of the region surrounding *GhPDS* target sites were identical in the two genomes (**Figure 2B**). Therefore, there were two binary vectors generated to be tested in hairy-root tissues.

### 2.3 CRISPR/Cas9-induced mutagenesis in cotton hairy-root tissues

**Figure 3:**
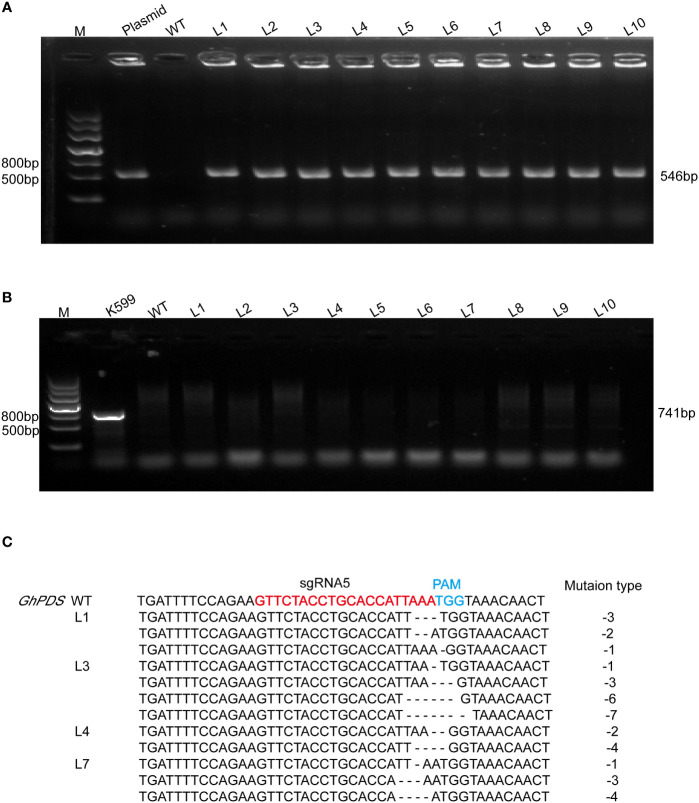
Analysis of mutations at sgRNA5 in R15 hairy roots. **(A)** Amplification products of *Cas9* from genomic DNA of R15 hairy roots using specific primer (**Supplementary Table S1**). M, 2-kb DNA marker; Plasmid, p2301-GhPDS-sgRNA5-CRISPR plasmid DNA (positive control); WT, wild-type hairy root DNA (negative control); L1-L10, ten independent cotton hairy roots. **(B)** Amplification products of *virB* from R15 hairy roots. K599, *A rhizogenes* strain K599 (positive control). **(C)** Hi-TOM sequencing analysis of sgRNA5 target site in positive hairy roots.

**Figure 4:**
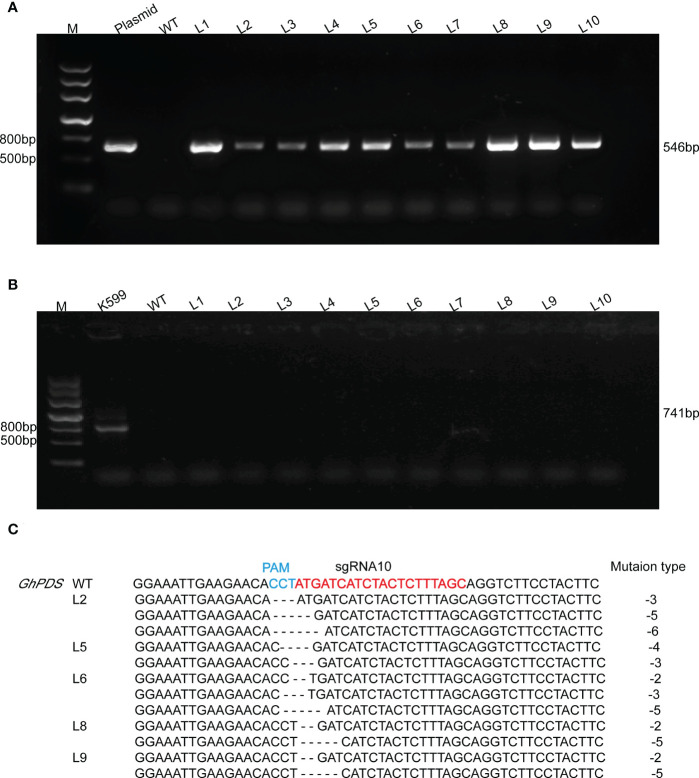
Analysis of mutations at sgRNA10 in R15 hairy roots. **(A)** Amplification products of *Cas9* from genomic DNA of R15 hairy roots using specific primer (**Supplementary Table S1**). M, 2-kb DNA marker; Plasmid, p2301-GhPDS-sgRNA10-CRISPR plasmid DNA (positive control); WT, wild-type hairy root DNA (negative control); L1-L10, ten independent cotton hairy roots. **(B)** Amplification products of *virB* from R15 hairy roots. K599, *A rhizogenes* strain K599 (positive control). **(C)** Hi-TOM sequencing analysis of sgRNA5 target site in positive hairy roots.

The validation of target sites was conveniently assessed using the developed hairy root system. *A. rhizogenes* strain K599 harboring the CRISPR plasmid was transferred into R15 apical bud. Ten hairy root lines were randomly selected for each sgRNA. The presence of *Cas9* gene in hairy root was detected by PCR using specific primers (**Figure 3A**, **4A**), and *virB* was not amplified in any lines for excluding *A. rhizogenes* contamination (**Figure 3B**, **4B**). These results demonstrated ten hairy root lines were positive. The two target regions in *GhPDS* were then amplified by PCR in ten positive hairy roots and the amplicons were sequenced by Hi-TOM. The results revealed that mutations were detected at sgRNA5 and sgRNA10 in hairy roots. Samples with target mutation ratio (target mutation reads/total reads containing target site) >1% were considered as edition occurred at the target site. Deletions ranging from -1 bp up to -7 bp in sgRNA5 were identified in 4 (L1, L3, L4, L7) out of 10 hairy root lines (**Figure 3C**) and deletions ranging from -2 to -6 bp in sgRNA10 were identified in 5 (L2, L5, L6, L8, L9) out of 10 hairy root lines (**Figure 4C**). These results demonstrated Cas9 and sgRNAs induced mutations could be detected in cotton hairy roots.

### 2.4 Targeted mutagenesis in transgenic cotton plants

**Figure 5:**
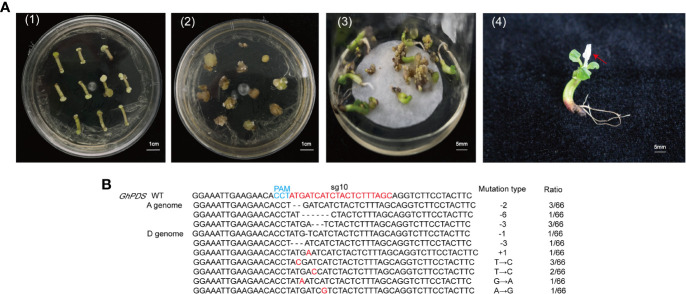
Mutations analysis of *GhPDS* in transgenic plants. **(A)** Generation of transgenic cotton plant *via A tumefaciens*-mediated transformation. (1) Induction of resistant calluses on selection medium; (2) embryonic calluses with different morphologies. (3) embryonic calluses were developed on regeneration medium. (4) regenerated transgenic cotton growing on regeneration medium. Red arrows indicated albino phenotype. **(B)** Sanger sequencing analysis of mutations at sgRNA10 target site in albino plantlet. The mutation types are present in the penultimate column. The number of amplicons giving mutant sequences out of the total clones are presented in the column to the right. -, deleted nucleotides; +, inserted nucleotides; →, substituted nucleotides.

Our method was efficient in selecting CRISPR/Cas9 target sites, but could not produce stable mutant plants. To test whether gene mutations detected in hairy roots could be detected in cotton, we performed stable transgenic transformation using *A. tumefaciens*-mediated hypocotyl transformation with the two CRISPR/Cas9 constructs. Some callus regenerated on selection medium about 4 months after transformation. One independent regenerated plant for the GhPDS-sgRNA10 target was obtained and showed an albino phenotype in the leaves (**Figure 5A**). However, no transgenic plants for the GhPDS-sgRNA5 were obtained. CRISPR/Cas9-induced mutations at sgRNA10 in the albino plantlet were analyzed by Sanger sequencing, the sequencing results showed successful editing events at sgRNA10 site in two homologous sequences of *GhPDS* were detected. A total of 66 clones were analyzed with 12.12% of them harboring deletions (-1, -2, -3, -6 bp), 1.52% containing insertions (+1 bp) and 10.60% containing substitutions. The frequency of deletion mutations was higher than that of insertions and substitutions (**Figure 5B**). Whereas, only deletion mutations were detected in the hairy roots. These results further demonstrate the feasibility of detection of sgRNAs using hairy root system in cotton.

## 3 Materials and methods

**Figure 6:**
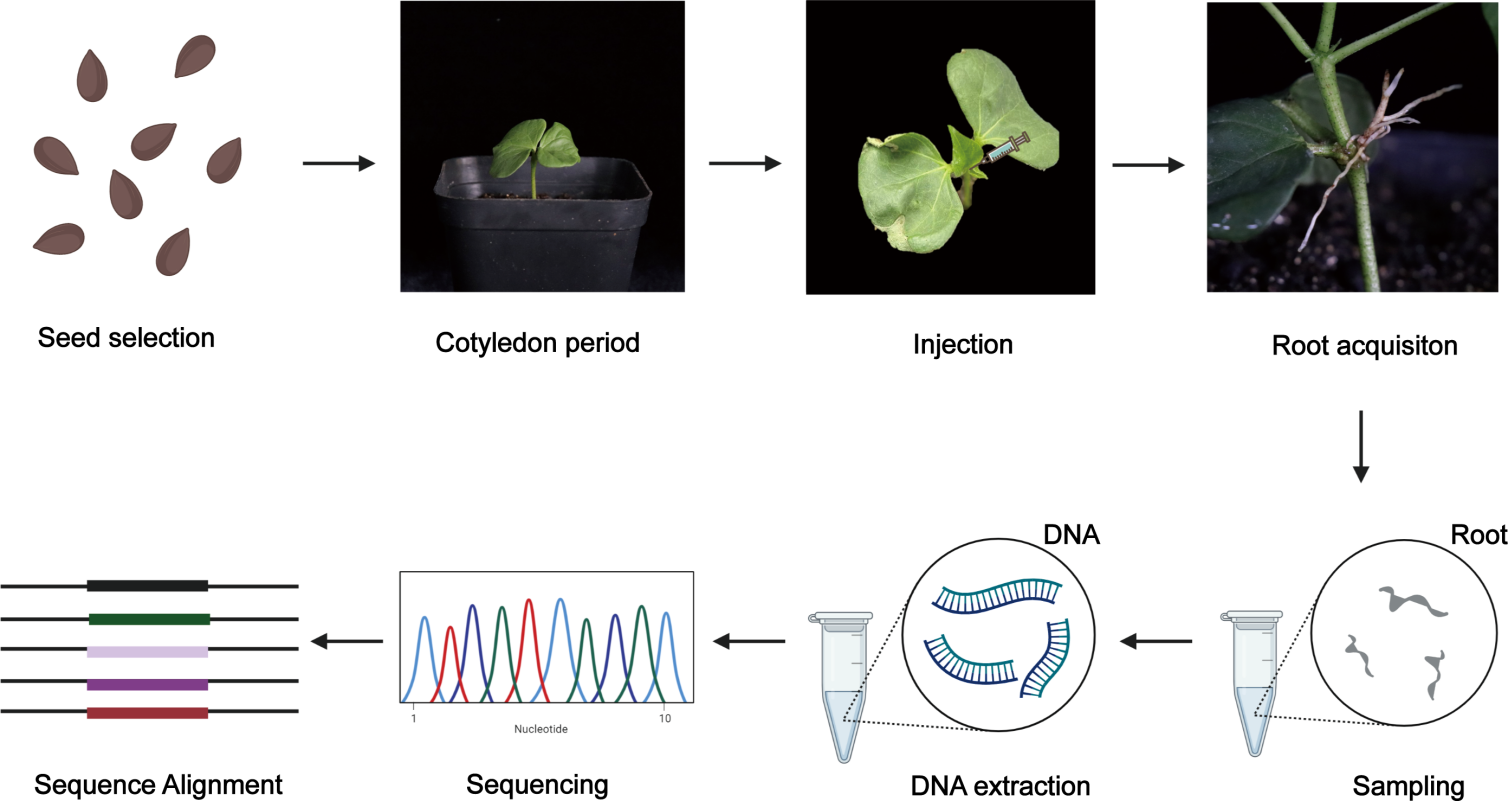
A workflow diagram of experiment. First, the plump cotton seeds were selected and planted in nutrient soil. Injection could be conducted when the first true leaf of cotton seedings unfurled. Then *A. rhizogenes* with OD_600_ 0.6–0.8 were prepared and used for injecting at the cotton apical bud. Hairy roots emerged within one month. Next, DNA was extracted from hairy roots and the positive hairy roots were detected using the specific primers. Fragments containing the target site were amplified with the specific primer and sequenced. Finally, alignment between sequencing results and original sequences was conducted.

A workflow diagram for the experiments is given in **Figure 6**.

### 3.1 Plants material and growth conditions

Four cotton (*Gossypium hirsutum* L.) cultivars (Coker 312 (R15), TM–1, Lumian–21 and Zhongmian–49) preserved in our laboratory were used in this study. These four cotton cultivars were planted in a growth chamber (16-h light/8-h dark; 100 µmol m^-2^ s^-1^ light intensity; 28°C; 70% humidity). Plants could be used for inducing hairy root when the first true leaf fully unfold (about 14 days after sowing). Coker 312 was used for stable transformation.

### 3.2 Construction of CRISPR/Cas9 vector

The CRISPR/Cas9 vectors were gifts from Jian-Kang Zhu (Feng et al., 2013). sgRNAs design according to standards mentioned previously (Li et al., 2017). Two sgRNAs (sgRNA5 and sgRNA10) DNA oligonucleotides were synthesized and annealed to generate two dimers. Two dimers were then cloned into the *Bbs I* site of pAtU6-26-SK to generate pSK-AtU6-sgRNA5 and pSK-AtU6-sgRNA10. These two pSK-AtU6-sgRNAs were cut with restriction enzyme *Kpn I/Sal I* (New England Biolabs, USA), and p35S-Cas9-SK was digested with restriction enzyme *Sal I/EcoR I*. Two fragments were assembled into pCAMBIA2301 using *KpnⅠ/EcoRⅠ* restriction digestion followed by ligation to generate the p2301-GhPDS-sgRNA5-CRISPR and p2301-GhPDS-sgRNA10-CRISPR constructs. The *kan* gene, driven by the *CaMV* 35S promoter of pCAMBIA2301, was used as selection marker for cotton stable transformation.

### 3.3 *Agrobacterium rhizogenes*-mediated transient transformation of cotton

*A. rhizogenes* strain K599 was used to deliver gene editing vector has been described (Fan et al., 2020). The binary vector was introduced into strain K599 purchased from WeiDi (Shanghai, China) using ‘heat shock’ procedure. *A. rhizogenes* strain K599 wild-type or carrying binary vectors were used for infection. Wild-type bacteria were cultured at 28°C with shaking at 180 rpm to different OD_600_ (0.4, 0.6, 0.8, 1.0). Afterwards, the bacterial suspension was used to inject the apical bud of 20 R15 plants with a concentration without centrifugation and resuspension. Plants were then grown in the growth chamber (16-h light/8-h dark; 28°C; 70% humidity) for 1 month, then hairy roots emerged and the number of plants with hairy roots was counted. The other three cotton cultivars were then injected in the same way. *A. rhizogenes* strain K599 harboring p2301-GhPDS-sgRNA5/10-CRISPR vector were cultured until the OD_600_ reached to 0.8 for injection as described above.

### 3.4 Genetic transformation of cotton

The cotton transformation was conducted according to the following method (Wang et al., 2017). Hypocotyl segments from R15 sterile seedings were immersed in the *Agrobacterium* strain LBA4404 (OD_600_ 0.4–0.5) for 5 min, then the infected hypocotyl fragments were co-cultured on MSB1 medium (Sigma, St. Louis, USA) for 48 h at 28°C in the dark. Next, the hypocotyls were transferred to MSB1 medium with 50 mg L^-1^ kanamycin and 500 mg L^-1^ cephalosporin for resistant calluses formation. The resistant calluses were grown on MSB2 medium for 4 months until embryogenic calluses (ECs) were obtained. ECs were then grown on MSB3 medium for 3–4 months. Finally, regenerated plants were transferred to soil.

### 3.5 Genomic DNA extraction and mutations analyses

DNA was extracted from hairy roots and leaves using the DNAsecure Plant Kit (TIANGEN, Beijing, China). Primers Cas9-F/R were designed based on *cas9* sequence to detect exogenous T-DNA, and primers VirB-F/R were designed based on *VirB* gene to detect *A. rhizogenes* contamination (**Supplementary Table S1**). The region spanning the target site was amplified using 2x Taq Plus Master Mix II (Vazyme, Nanjing, China) with the specific primer pair (**Supplementary Table S1**). The amplicons were sequenced by high-throughput (HiTOM) sequencing platform (Liu et al., 2019) and Sanger sequencing.

### 3.6 Statistical analyses

Samples were collected from more than three technical replicates. The number of plants with hairy roots data were analyzed using SPSS software (IBM, Armonk, NY, USA). Analysis of variance was used to compare the statistic difference based on Tukey’s HSD test at significance level of p < 0.05.

## 4 Discussion

*A. rhizogenes* K599 strain was able to induce hairy roots has been reported earlier in cotton (Che et al., 2019) and other plants (Wei et al., 2016; Aggarwal et al., 2018; Bazaldúa et al., 2019). Previous studies have indicated that some experimental steps need to be optimized for effective utilization of transformation ability of *A. rhizogenes.* In this study *A. rhizogenes* K599 strain could infect cotton apical bud and induced the infected cells to form hairy roots at 30% efficiency when bacteria were cultured until the OD_600_ reached to 0.8 (**Figure 1A**) and the procedure could be completed within one month. Previously, the procedure of hairy root transformation needs to be done in a sterile environment (Verma et al., 2009; Cui et al., 2020), whereas our method needs no sterile conditions. Compared with *A. tumefaciens*-mediated transformation, *A. rhizogenes-*mediated hairy root transformation is genotype-independent (Hosseini et al., 2017; Aggarwal et al., 2018; Gomes et al., 2019). Our study showed that *A. rhizogenes* K599 strain could induce hairy roots from four cotton cultivars and hairy root induction frequency showed no significant difference between them (**Figures 1B, C**).

Plant mutants are particularly important for studying gene functions and molecular mechanisms, and the CRISPR/Cas9 system provides an efficient method to induce mutations in target genes for functional research and genetic improvement of crops. CRISPR/Cas9 technology has been successfully applied in many crops for genome engineering, including cotton (Farboud and Meyer, 2015; Gao et al., 2017; Gao et al., 2020). Multiple sgRNAs are designed to ensure that the target gene is edited. *A. tumefaciens*-mediated genetic transformation procedure of cotton is time-consuming and inefficient. Therefore, it is essential to select efficient sgRNAs to reduce the workload. Hairy root system has been used for genome editing of various plant species, such as *Medicago truncatula* (Zhang et al., 2020), cucumber (Nguyen et al., 2021), oilseed rape (Jedličková et al., 2022). For cotton, the hairy root system has been applied for studying promoter activity (Kim et al., 2011), secondary metabolites production (Verma et al., 2009; Kim et al., 2011), plant generation (Cui et al., 2020), cotton-nematode interaction (Wubben et al., 2009), however, it has not been used for genome editing. Cotton is an allotetraploid and A and D genomes have a high content of repetitive DNA (Li et al., 2015). It is necessary to mutate multiple homoeoalleles for gene functional studies. Hairy root system is useful to assess the activity of sgRNA. In our study, two sgRNAs (sgRNA5 and sgRNA10) targeting *GhPDS* in A and D genomes were designed and the deletions were detected at the both sites in hairy roots (**Figures 3C**, **4C**), the most common mutations induced by CRISPR/Cas9 were deletions, which is consistent with the results reported in rice, *Arabidopsis*, tobacco and other species (Nekrasov et al., 2013; Li et al., 2014; Xu et al., 2014; Zhou et al., 2014; Fan et al., 2015; Sun et al., 2015). Most practical applications of CRISPR/Cas9 system need obtained transgenic plants when studying important agronomic traits (Gao et al., 2017). The mutations in sgRNA10 in transgenic hairy roots were also observed in transgenic cotton which showed an albino phenotype (**Figure 5B**). This rapid and simple method could rapidly validate CRISPR/Cas9 sgRNA targets and reduce the workload of cotton stable transformation.

The hairy roots induced by *A. rhizogenes* are easy to cultivate and grow rapidly and have been widely used in the production and extraction of active ingredients of medicinal plants, such as ginseng (Fukuyama et al., 2012; Kochan et al., 2013), *Artemisia annua* (Patra and Srivastra, 2014), *Salvia miltiorrhiza* (Kai et al., 2011; Wei et al., 2019), *Camptotheca* acuminata (Lorence et al., 2004). Therefore, hairy root system provides an efficient method for secondary metabolites research in cotton (Verma et al., 2009). Compared with hairy root from cotyledon and hypocotyl (Verma et al., 2009; Cui et al., 2020), transgenic hairy root and composite plant could be used to study the transport of small signaling molecules like peptides and metabolites between roots and other parts of the plant (Che et al., 2019). Altogether, this method provides an opportunity to study root biology and gene function studies.

## 5 Conclusion

In this study, a simple and effective method under non-sterile conditions for cotton hairy root induction and transformation was developed for four cotton cultivars. The targeting capability of sgRNA could be evaluated successfully using this method. The results provided a potential tool for gene expression and gene editing studies in cotton.

## Data availability statement

The original contributions presented in the study are included in the article/**Supplementary Material**. Further inquiries can be directed to the corresponding authors.

## Author contributions

Conceptualization, LZ and YW. Methodology, PW. Software, CW. Validation, LZ, YW and JW. Formal analysis, LZ. Investigation, JW. Resources, CW. Data curation, PW. Writing original draft preparation, LZ. Writing review and editing, LZ and YW. Visualization, JW. Supervision, HC. Project administration, XW. Funding acquisition, XW and HC. All authors contributed to the article and approved the submitted version.
